# Developmental Constraints on Learning Artificial Grammars with Fixed, Flexible and Free Word Order

**DOI:** 10.3389/fpsyg.2017.01816

**Published:** 2017-10-17

**Authors:** Iga Nowak, Giosuè Baggio

**Affiliations:** ^1^Institute of Neuroscience and Psychology, University of Glasgow, Glasgow, United Kingdom; ^2^International School for Advanced Studies, Trieste, Italy; ^3^Language Acquisition and Language Processing Lab, Department of Language and Literature, Norwegian University of Science and Technology, Trondheim, Norway

**Keywords:** language learning, cognitive development, grammar, syntax, artificial grammars

## Abstract

Human learning, although highly flexible and efficient, is constrained in ways that facilitate or impede the acquisition of certain systems of information. Some such constraints, active during infancy and childhood, have been proposed to account for the apparent ease with which typically developing children acquire language. In a series of experiments, we investigated the role of developmental constraints on learning artificial grammars with a distinction between shorter and relatively frequent words (‘function words,’ F-words) and longer and less frequent words (‘content words,’ C-words). We constructed 4 finite-state grammars, in which the order of F-words, relative to C-words, was either *fixed* (F-words always occupied the same positions in a string), *flexible* (every F-word always followed a C-word), or *free*. We exposed adults (*N* = 84) and kindergarten children (*N* = 100) to strings from each of these artificial grammars, and we assessed their ability to recognize strings with the same structure, but a different vocabulary. Adults were better at recognizing strings when regularities were available (i.e., fixed and flexible order grammars), while children were better at recognizing strings from the grammars consistent with the attested distribution of function and content words in natural languages (i.e., flexible and free order grammars). These results provide evidence for a link between developmental constraints on learning and linguistic typology.

## Introduction

Humans are highly flexible and efficient learners, yet their capacity to acquire information is constrained at different levels by the organization of cognitive and perceptual systems (Shepard, 2001; Krakauer and Mazzoni, 2011). Typically developing children can learn several languages with striking ease, exploiting a variety of learning mechanisms, from implicit statistical learning to forms of cultural learning (Newport, 1990; Tomasello, 2003; Yang, 2003; Ambridge and Lieven, 2011; Chater et al., 2015). A classic argument is that children would be unable to acquire languages, if the space of possible target grammars was not (initially) restricted, e.g., by the relevant learning algorithms (Chomsky, 1965; Pinker, 1984). Grammars which conflict with such constraints would be more difficult (or impossible) to learn. Current debates focus on the *scope* (to what aspects of language, e.g., syntax, would learning constraints apply?) (Wilson, 2006; St. Clair et al., 2009; Culbertson et al., 2012), on the *nature* (are learning constraints specific to language, or is language learning constrained by other domains, e.g., by auditory perception?) (Endress et al., 2009; Fava et al., 2011), and on the *timing* of learning constraints (do they appear early in infancy and wane later in childhood, or could they also exert their effects throughout the lifespan?) (Newport, 1990; Birdsong, 1992; Hudson Kam and Newport, 2005). Here, we investigate two related issues of *scope* and *timing*: the present study does not address the *nature* and *origins* of learning constraints.

The *scope* issue addressed here is whether learning constraints apply to arguably the most general distinction between lexical categories-*function* and *content words*. This distinction is closely related to, but does not coincide with, the distinction between *open* and *closed class words*. These word classes are defined with reference to the likelihood (or the historical frequency) with which languages acquire (or have acquired) new such elements. For example, in many languages of the world, nouns and verbs are constantly being added to the language’s dictionary (the noun and verb classes are said to be ‘open’), while determiners and prepositions are added at much slower rates (they are ‘closed’ classes). Instead, function and content words are defined by a cluster of morpho-syntactic, semantic or statistical properties. Syntactically, function and content words correspond to different grammatical categories (i.e., parts of speech), in ways that may differ across languages. For example, in English, function words are prepositions, determiners, connectives etc., while content words are verbs, nouns, adjectives, adverbs etc. Semantically, function words determine the *logical form* of a sentence (logical connectives, quantifiers etc.) and have important pragmatic properties (e.g., they can trigger implicatures). Content words, instead, contribute more to the *lexical and conceptual content* of sentences, and less to their logical form. Statistically, function words are shorter and more frequent than content words (Miller et al., 1958; Grimshaw, 1981). Word length and frequency are clearly associated with the distinction between content and function words, but do not *constitute* the difference: the real differences are syntax and semantics.

In the absence of syntactic or semantic information about new words, children may actively use word length or frequency cues to begin to crack the syntactic structure of the input. The ability to classify new words as function words (shorter, more frequent) or content words (longer, less frequent) may facilitate subsequent learning of the grammatical category to which each new word belongs. Thus, the initial identification of function and content words in the input may be a first step to solving the ‘syntactic bootstrapping’ problem. For example, in languages like English, learners may use very high-frequency morphemes, such as ‘the,’ as anchor points, and observe what words co-occur with them (Valian and Coulson, 2002). This distributional analysis would allow children to learn constraints on the structure of noun phrases. In general, the relative position of function and content words in input strings may provide information about the order of certain types of constituents, thus facilitating learning by infants, older children or adults (Braine, 1966; Morgan et al., 1987; Valian and Coulson, 2002; Gervain et al., 2008; Bell et al., 2009; Hochmann et al., 2010; Gervain et al., 2013). Conversely, a grammar may become difficult (or impossible) to learn if function words are vanishingly rare or absent from the input. Previous work in this field has made use of artificial grammars, which lend themselves well to studying situations in which learning is influenced by word frequency, length or position in strings, as with the distinction between function and content words.

The *timing* question raised here is whether *developmental constraints* exist that make it easier for children (but not for adults) to learn grammars in which function and content words occupy *typologically plausible positions* in strings (i.e., grammars where the order of those words is *flexible* or *free*), as compared to grammars in which words are implausibly placed in strings (i.e., function or content words occupy *fixed* positions in a string’s linear order). At the most abstract level of structural description, the order of function words and content words across natural languages is *free*. If one were to replace all function words in a language (or a corpus) with one F symbol, and all content words with one C symbol, then one would obtain binary strings (‘FCFCCF…’) embodying few or no constraints on the relative position of those symbols. If, however, one considers suitable *fragments* of the language, then one can find systematic *local patterns*: e.g., determiners precede nouns in most European languages; natural languages differ as to whether certain function words are prepositions or postpositions; etc. However, no human language has rules for placing function and content words in *fixed positions* (e.g., as first and last) in strings. Languages that embody such rules, and a linear order of constituents more generally, are empirically unattested, and either extremely unlikely or, as some have suggested from the standpoint of generative syntax, impossible (Moro, 2016). Research has shown that behavioral and neural measures are sensitive to the distinction between plausible and implausible (or possible *vs* impossible) grammars and constituent orders (Tettamanti et al., 2002, 2008; Musso et al., 2003), and that learning in adults and children can reflect typological patterns (Saffran et al., 1996, 1999; St. Clair et al., 2009; Culbertson, 2012). The existence of typological constraints on learning is thus well motivated theoretically and empirically. The timing question posed here is whether those are *developmental* constraints or not: i.e., whether learning in children, but not in adults, is affected by different kinds of rules (plausible *vs* implausible) for placing function and content words in strings.

**FIGURE 1:**
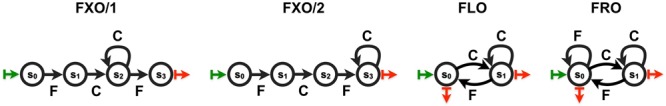
State-transition diagrams of the non-deterministic finite-state automata generating strings from the grammars used in the present study. Circles are states, and black arrows are transitions between states. The initial and final states are indicated by green and red arrows, respectively. At every transition, a symbol from the F or C types is generated. All strings used in the present study had a minimum length of 3 words: we considered only (a subset of) the strings that can be generated after 3 or more transitions.

We designed 4 artificial finite-state grammars with 2 symbols: ‘F’ and ‘C.’ Such grammars are inadequate for capturing even the basic properties of phrase structures, but what we aim to describe here are *abstract alternation patterns* of function (‘F’) and content (‘C’) words. We selected 4 string types, generated by each (non-deterministic) finite-state automaton that represents one grammar (Levelt, 2008; **Figure 1** and **Table 1**). In 2 grammars, word order is strictly *fixed*: in FXO/1, F-words occur only as the first and last symbols in a string, all other symbols being C-words; in FXO/2, F-words occur only as the first and the third symbols in a string, all other symbols being C-words. In the other 2 grammars, ordering is less constrained: in the *flexible order* grammar FLO, F-words can occur in any position in a string, but must follow a C-word, while in the *free order* grammar FRO, F- and C-words can occupy any position in a string. Therefore, in FXO grammars, the order of F- and C-words is *fully constrained*, in FLO it is *partly constrained*, and in FRO it is *unconstrained*.

**Table 1:** Experimental strings from each of the four artificial grammars examined here.

Grammar	String types	*K*	*E*	FC;CF	FC + CF	FF;CC	FF + CC	S
FXO/1	FCF	1.585	0.918	1;1	2	0;0	0	2
	FCCF	1.5	1	1;1	2	0;1	1	3
	FCCCF	1.393	0.971	1;1	2	0;2	2	4
	FCCCCF	1.293	0.918	1;1	2	0;3	3	5
	*Total*			*4;4*	*8*	*0;6*	*6*	
FXO/2	FCF	1.585	0.918	1;1	*2*	0;0	0	2
	FCFC	1.5	1	2;1	3	0;0	0	3
	FCFCC	1.393	0.971	2;1	3	0;1	1	4
	FCFCCC	1.723	0.918	2;1	3	0;2	2	5
	*Total*			*7;4*	*11*	*0;3*	*3*	
FLO	CFC	1.585	0.918	1;1	*2*	0;0	0	2
	CFCF	1.5	1	1;2	3	0;0	0	3
	CFCFC	1.393	0.971	2;2	4	0;0	0	4
	CFCFCC	1.393	0.971	2;2	4	0;1	1	5
	*Total*			*6; 7*	*13*	*0;1*	*1*	
FRO	FCC	1.585	0.918	1;0	1	0;1	1	2
	CFFC	1.5	1	1;1	2	1;0	1	3
	CCFCF	1.393	0.971	1;2	3	0;1	1	4
	FCCFCC	1.723	0.918	2;1	3	0;2	2	5
	*Total*			*5;4*	*9*	*1;4*	*5*	

*K is the Kolmogorov complexity and E is the Shannon entropy of string types. The frequencies of transitions between word types (FC, CF, FF, CC) in each string type are shown, with sums of transition frequencies between word types (FC + CF), within word types (FF + CC), and across all transition types (S = FC + CF + FF + CC). More information on the exposure and test conditions is provided in **Table 2**. Sample strings are provided as Supplementary Material.*

From these 16 string types, we constructed experimental strings with two types of pseudowords: frequent monosyllabic F-words (e.g., ‘ri,’ ‘om,’ ‘of,’ ‘cu,’ ‘en,’ ‘ba’), and less frequent bisyllabic C-words (e.g., ‘bori,’ ‘depo,’ ‘alfon,’ ‘sasne’), intended to mimic function and content words in natural languages, respectively (see section “Materials and Methods” and Supplementary Material for more details). In our stimulus sets, each F-word occurred 6 times more frequently than any C-word (Valian and Coulson, 2002). We exposed 4 groups of young adults and 4 groups of kindergarten children, whose first languages were German, Italian or Polish, to strings from each grammar (e.g., FLO), and we then tested them for recognition of strings from the same grammar *vs* strings from a grammar they had not been exposed to (e.g., FXO/1; **Table 2**) (Reber, 1989; Crain and Thornton, 2000; Perruchet and Pacton, 2006; Rohrmeier et al., 2012). The test strings had the same structure as the exposure strings, but were built using different words. The same apparatus, stimuli, task and procedure were used for adults and children; feedback was given, as to whether their response in each test trial was correct or incorrect.

**Table 2:** Exposure and test conditions across experiments and groups.

Experiment	Group	*N*	Exposure	Strings/type (total)	Test	Strings/trial (total)
1	Adults	21	FXO/1	12 [144]	FXO/1, FLO	3, 3 (24, 24)
	Adults	21	FLO	12 [144]	FXO/1, FLO	3, 3 (24, 24)
2	Adults	21	FXO/2	12 [144]	FXO/2, FRO	3, 3 (24, 24)
	Adults	21	FRO	12 [144]	FXO/2, FRO	3, 3 (24, 24)
3	Children	22	FXO/1	12 [144]	FXO/1, FLO	3, 3 (24, 24)
	Children	22	FLO	12 [144]	FXO/1, FLO	3, 3 (24, 24)
4	Children	28	FXO/2	12 [144]	FXO/2, FRO	3, 3 (24, 24)
	Children	28	FRO	12 [144]	FXO/2, FRO	3, 3 (24, 24)

*All string types (4 per grammar; **Table 1**) were used with equal frequency. In each exposure set, 12 strings per type (4 types; **Table 1**) were played, and each string was repeated three times non-successively (total 144 strings). In each test set, 3 strings per grammar per trial were played, i.e., 24 strings per grammar, or a total of 48 strings.*

Our predictions are as follows. Young adults are expected to be highly adept at detecting complex patterns in temporal sequences, using algorithms or heuristics largely unavailable to most kindergarten children (i.e., counting, phonological and linguistic awareness, full attentional control etc.). Patterns of alternation between short and frequent (F-words) and long and infrequent (C-words) constituents should be fairly obvious to adults, for the grammars where those patterns exist (Reber, 1989; Gómez and Gerken, 2000; Perruchet and Rey, 2005; de Vries et al., 2008): we therefore expect that the only condition in which adults fail is FRO, where regularities are absent. The results should be different for children, partly owing to the developmental constraints shaping cognition around kindergarten age (Slobin and Bever, 1982). Grammar learning in children may be more sensitive to the constraints that parallel typological patterns (Hudson Kam and Newport, 2009; Culbertson et al., 2012; Culbertson, 2012; Fedzechkina et al., 2012): grammars that violate such patterns should be more difficult to learn, and less likely to be acquired by new generations of language users (Culbertson, 2012). In no natural language are function words positioned according to the placement rules of F-words in FXO/1 and FXO/2. Children may find these grammars harder to learn. In contrast, FLO and FRO instantiate plausible patterns of occurrence of function and content words in natural languages. Accordingly, children are expected to find these grammars easier to learn. More specifically, we predict the following response patterns during string recognition at test. Adults in the FXO/1, FXO/2 and FLO groups should perform above chance, whereas adults in the FRO group should be at chance. Moreover, children in the FLO and FRO groups should perform above chance, while children in the FXO/1 and FXO/2 groups should be at chance. If learning constraints are universal, these effects should *not* be modulated by the native language (L1) of adults and children. Hence, we tested participants from different language backgrounds, i.e., Italian, German and Polish. We also aimed to determine to what extent children would learn during test, possibly as a result of feedback: if that is the case, performance should improve over test trials in the children groups trained on the FLO and FRO grammars, but not in the groups trained on the FXO/1 and FXO/2 grammars, where instead performance is predicted to remain at chance levels.

## Materials and Methods

### Participants

We tested 42 adults in Experiment 1 (22 Italian and 20 Austrian, 29 women, mean age 22.44 years, range 19–35) and 42 adults in Experiment 2 (22 Italian and 20 Polish, 31 women, mean age 20.66, range 19–27). Written informed consent was obtained from all participants, who were paid for taking part in the study. We tested 44 children in Experiment 3 (22 Italian and 22 Austrian, 23 girls, mean age 4.5, range 3–6) and 56 children in Experiment 4 (28 Italian and 28 Polish, 32 girls, mean age 4.8, range 3–6). These groups were disjoint, independent samples, and were matched for age, gender and education level. Only adults and children who had grown up in monolingual families, in which parents spoke either Italian, German or Polish, were included in the study. All adults had completed upper secondary level education. In Experiments 3 and 4, 5 and 8 children, respectively, were excluded from further analyses. These children either showed lack of interest in the task, and decided to quit before the experiment was completed, or showed lack of understanding of the task, as evidenced by their failure to respond to every trial at test. Incomplete data sets were generated in these cases, therefore the decision to exclude these 13 children from further analyses could be taken at test. Children were recruited from 4 kindergartens in Italy, Austria and Poland. All and only the children of parents who returned a signed informed consent sheet took part in the study. The experiment was approved by the Ethics Committee at the International School for Advanced Studies (SISSA) in Trieste. The study was carried out in accordance with the approved protocol.

### Materials

We selected 4 string types generated by each grammar (**Figure 1** and **Table 1**) as a basis for constructing F- and C-word strings (Supplementary Material). Across grammars, strings were matched in length (from 3 to 6 words) and in the number of F-words (2) and C-words (1–4) in each string. Moreover, the complexity of *binary string types* of a given length, measured by Kolmogorov complexity (Lempel and Ziv, 1976) and Shannon entropy (Shannon, 1948), was comparable across grammars (**Table 1**). The complexity of *actual strings* of a given length, where Fs and Cs are replaced with pseudowords, is trivially the same across grammars: each word counts as a different symbol in a string. In our stimuli, no F- or C-word was ever repeated in a string. Therefore, *n*-gram frequency, for strings of a given length, is trivially matched across grammars. In a further control study, we calculated the Kolmogorov complexity (*K*) and Shannon entropy (*E*) of *binary string types* of F and C symbols, of length up to 12 words, exceeding the maximum length of experimental strings (6) by 6, to assess whether the complexity of string types in different grammars diverged with increasing string length. That was not the case. Comparing the averages of *K* and *E* across string types between the grammar pairs in each experiment (FXO/1 vs. FLO and FXO/2 vs. FRO) using Wilcoxon rank sum tests yielded no effects (FXO/1 vs. FLO, K: W = 29.5, *p* = 0.129, E: W = 42, *p* = 0.568; FXO/2 vs. FRO, K: W = 39.5, *p* = 0.447, E: W = 49, *p* = 0.97).

Experimental stimuli were created by substituting all F and C symbols in each string generated by a grammar (**Table 1**) with artificial F- or C-words (see below). We constructed separate *exposure* and *test* sets. In the *exposure sets*, each F-word occurred with a frequency 6 times higher than any C-word, in different positions in each string (**Figure 1** and **Table 1**). The *test sets* differed from the exposure sets in that different actual F- and C-words were used. In every experiment, *string structure, and the grammars generating the exposure and test strings, were the same*, while their ‘vocabularies’ differed. A total of 132 C-words were used in the exposure sets for the FRO and FLO grammars, and 120 for FXO/1 and FXO/2, whereas 66 C-words, and respectively 60 in FXO/1 and FXO/2, were used in the test sets. All F-words in the exposure and test sets were monosyllabic, either consonant-vocal or vocal-consonant pairs, and all C-words were bisyllabic. All syllables were randomly drawn from the syllabic repertoires of Italian, German and Polish, but none of the words used in the stimuli were actual Italian, German or Polish words. Moreover, none of the words included overt violations of Italian, German or Polish phonotactics. These phonological and structural features of the strings arise from an effort to satisfy multiple constraints simultaneously, i.e., to match (a) the *structural complexity* of strings of a given length across grammars, (b) the *number* of F- or C-words across strings and grammars, (c) the *frequency* of F- and C-words across grammars, and overall, (d) *string length*, and (e) the *phonological and phonotactic complexity* of words across strings and grammars. In order to be able to attribute to the *formal structure of strings* any observed differences in recognition performance or learning by children and adults, one should hold all these stimulus features largely constant across grammars. Examples of the strings used in the experiments are provided as Supplementary Material. A trained female phonetician was recorded while she read each pseudoword in a natural animated voice. A single recorded audio token for each word was used to generate the strings, collating together audio clips for each word in a string. The auditory offset of one word and the onset of the next word were separated by 200 ms of silence (Marchetto and Bonatti, 2013). Audio files were normalized to a mean intensity of 60 dB, and were played at the same volume in all sessions. Adjacent strings were separated by 1s of silence.

### Apparatus

To heighten the children’s interest in the stimuli, we used a colorful puppet theater (∼150 cm width, 50 cm depth, 50 cm height), with two soft cloth puppets: a flower and an elk (Crain and Nakayama, 1987). All strings were presented auditorily, via two loudspeakers placed on the theater’s stage to the left and right of its vertical symmetry axis, invisible to the participants. A curtain along the front side of the theater, and facing participants, could be closed, so as to render the stage, and the experimenter behind it, invisible to participants. On the theater’s stage were two boxes with lids. The boxes were visible to the participants only when the curtain was fully open. Each box was placed in front of (and hiding) a loudspeaker.

### Procedure

Each session (one participant) consisted of an *exposure phase*, followed by a *test phase*. We employed a between-subjects design, where participants were randomly assigned before exposure to one of two counterbalanced grammar conditions: FXO/1 or FLO in Experiments 1 (adults) and 3 (children), FXO/2 or FRO in Experiments 2 (adults) and 4 (children), as detailed in **Table 2**. The exposure and test procedures were exactly the same for children and adults.

#### Exposure Phase

Participants were introduced to puppet A (‘Flower’). They were told that he was from a distant land, and spoke a foreign language. They were urged to listen to him carefully, as they would be asked questions concerning his language later. Participants were then exposed to 48 auditory strings from the exposure set, sequentially with no breaks, delivered through both loudspeakers simultaneously. Each string was presented exactly three times non-consecutively, for a total of 144 strings. The exposure phase lasted about 5 min, during which puppet A was always visible on stage. The exposure phase was immediately followed by the test phase.

#### Test Phase

We implemented a 2-alternative forced choice (2AFC) task to assess the participants’ ability to *recognize novel test strings with the same structure as the strings they had heard during the exposure phase, but different ‘vocabulary.’* Participants were first introduced to puppet B (‘Elk’). They were told that puppet B speaks a different language than puppet A (‘Flower’), and that both puppets now want to play a game with them: each puppet will hide inside one box, and they (participants) would have to listen to strings coming from each box, and guess where the puppets are hiding. The curtain was then closed, and the puppets A and B were placed inside each box (call them box A and B, respectively). This process, and the experimenter carrying it out, were invisible to the participants. After closing the lid of each box, the curtain was opened. Participants listened to sequences of 3 strings coming from one box, followed by 3 strings from the other box (6 strings per trial). Strings coming from box A, where puppet A was hiding, had the same structure as the strings that participants had heard during exposure (e.g., FLO), whereas strings from box B were generated by the alternative grammar (e.g., FXO/1 in Experiment 1). The position of the boxes A and B, in which puppets A and B, respectively, were hidden, was randomized across participants and trials. After a 6-string set (a trial) was played, the experimenter asked the participant in which box puppet A was hiding. The participant responded by pointing to or by verbally referring to either of the boxes: no other types of answer were deemed valid. First, the experimenter opened the box chosen by the participant, showing its content to them. Next, the other box was opened, and its content was shown to the participant. This procedure was repeated for 8 consecutive test trials, lasting about 12 min.

### Data Analysis

**FIGURE 2:**
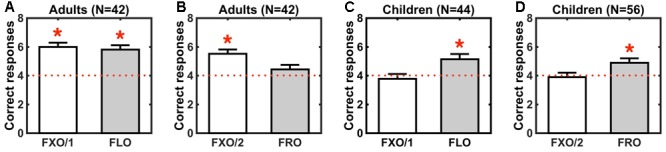
String recognition performance (average correct trials) in adults exposed to a fixed order grammar (FXO/1) or a flexible order grammar (FLO) in Experiment 1 **(A)** and to a fixed order grammar (FXO/2) or a free order grammar (FRO) in Experiment 2 **(B)**, and in children exposed to the same grammars in Experiments 3 **(C)** and 4 **(D)**. Different colors of bars (white or gray) correspond to different groups of participants, exposed to different grammars. Error bars denote standard errors of the mean. Red dotted lines show chance level (4 correct trials out of 8). Red asterisks indicate statistically significant effects relative to chance (**Table 3**).

Three sets of data analyses were performed. First, we conducted an omnibus ANOVA, including data from all children and adult groups, and using Group (adults or children), Grammar (FXO/1, FXO/2, FLO or FRO) and L1 (Italian, German or Polish) as between-subjects factors. We constructed two separate models, with performance (correct responses in the test phase) and learning (the difference in the number of correct responses between the first and the second halves of the test phase) as dependent variables. We then conducted further ANOVAs for adults and children separately, using the same factors as above (Grammar and L1), and performance as a dependent variable. Second, correct responses in the test phase were compared to chance level (4 correct responses in 8 trials), in each grammar group separately, using one-sample Wilcoxon signed rank tests (**Table 3** and **Figure 2**). Third, linear models, one per grammar type, with Trial (1–8) as predictor of performance, were used to determine how the number of correct responses changes in the course of the test phase. Statistical analyses were carried out using R (R Development Core Team, 2008). Effect sizes (Cohen’s *d* and η^2^) regression coefficients, test-statistics and *p*-values are reported.

**Table 3:** String recognition performance in adults trained on a fixed order grammar (FXO/1) or a flexible order grammar (FLO) in Experiment 1 and to a fixed order grammar (FXO/2) or a free order grammar (FRO) in Experiment 2, and in children trained on the same grammars in Experiments 3 and 4.

Experiment	Group	*N*	Grammar	M (SEM)	*d*	*V*	*p*	Figure
1	Adults	21	FXO/1	6 [0.285]	1.534	206	<0.001	2A
	Adults	21	FLO	5.81 [0.298]	1.326	186	<0.001	2A
2	Adults	21	FXO/2	5.52 [0.29]	1.148	161	<0.001	2B
	Adults	21	FRO	4.43 [0.313]	0.299	102	0.221	2B
3	Children	22	FXO/1	3.77 [0.335]	-0.145	47.5	0.772	2C
	Children	22	FLO	5.14 [0.362]	0.669	159	0.009	2C
4	Children	28	FXO/2	3.89 [0.306]	-0.065	93.4	0.676	2D
	Children	28	FRO	4.89 [0.306]	0.511	187.5	0.011	2D

*Means (SEMs) of correct responses over trials are reported, with the results of one-sample Wilcoxon signed rank tests against chance level (4) and effect sizes (d).*

## Results

**FIGURE 3:**
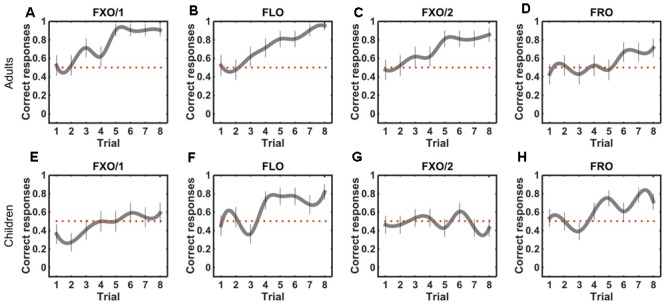
String recognition performance (correct responses over trials) in adults exposed to a fixed order grammar (FXO/1) or a flexible order grammar (FLO) in Experiment 1 **(A,B)** and to a fixed order grammar (FXO/2) or a free order grammar (FRO) in Experiment 2 **(C,D)**, and in children exposed to the same grammars in Experiments 3 **(E,F)** and 4 **(G,H)**. Different charts correspond to different groups of adults and children, trained on different grammars. Error bars denote standard errors of the mean. Red dotted lines show chance level (0.5).

Our results show that both children and adults can recognize strings from the flexible order grammar (FLO). However, performance differs between adults and children in the fixed and free order grammars, i.e., only adults recognize strings from the fixed order grammars (FXO/1 and FXO/2), and only children recognize strings from the free order grammar (FRO) (**Figure 2**). Recognition performance changes over test trials following the same pattern: it improves (from chance to above-chance levels), in children and adults, with the flexible order grammar (FLO), but it increases with the fixed order grammars (FXO/1 and FXO/2) only in adults, and it improves more steadily with the free order grammar (FRO) in children than in adults (**Figure 3**).

Before we investigated the behavior of adults and children separately, we tested for differences between groups by means of an omnibus ANOVA. The largest effects on performance (number of correct trials) were found for the factor Group [η^2^= 0.092, *F*(1,168) = 22.02, *p* < 0.0001] and the interaction of Group and Grammar [η^2^= 0.092, *F*(3,168) = 7.33, *p* = 0.0001]. Other main effects and interactions were smaller [η^2^< 0.04, *p* > 0.01]. Likewise, the largest effects on the learning measure (performance change over trials) were observed for Group [η^2^= 0.036, *F*(1,168) = 7.43, *p* = 0.007] and Group × Grammar [η^2^= 0.046, *F*(3,168) = 3.17, *p* = 0.026]. Thus, children and adults perform differently in the string recognition task on average, and moreover their performance changes (or fails to change) differently over trials. These effects were further explored in group-specific analyses.

### Adults

In Experiment 1, adult participants successfully recognized strings from the FXO/1 and FLO grammars, after they had been exposed to strings from each grammar: in both cases, performance was significantly above chance (**Figure 2A** and **Table 3**). In Experiment 2, adult participants could recognize strings from the FXO/2 grammar when they had been trained on it, but they were unable to recognize FRO strings following exposure to the FRO grammar (**Figure 2B** and **Table 3**). A between-groups ANOVA resulted in a main effect of Grammar on performance [η^2^= 0.174, *F*(3,76) = 6.196, *p* = 0.0008], not modulated by the first language (L1) of participants [Grammar × L1: η^2^= 0.013, *F*(2,76) = 0.7, *p* = 0.5]. In line with our predictions, adults recognize strings whenever an underlying structural pattern can be detected (in fixed and flexible order grammars), but they fail when regularities are absent (in the free order grammar).

We analyzed changes in performance during the test phase. We found that the number of correct responses increased over trials in all adult groups [FXO/1: *R*^2^= 0.112, η^2^= 0.117, *F*(1,166) = 21.98, *p* < 0.0001; FXO/2: *R*^2^= 0.077, η^2^= 0.082, *F*(1,166) = 14.85, *p* = 0.0001; FLO: *R*^2^= 0.121, η^2^= 0.126, *F*(1,166) = 24, *p* < 0.0001; FRO: *R*^2^= 0.029, η^2^= 0.034, *F*(1,166) = 5.917, *p* = 0.016; **Figures 3A–D**). In all cases, performance was at chance level in the first two trials of the test phase (FXO/1: V = 49, *p* = 0.812; FXO/2: V = 27.5, *p* = 1; FLO: V = 39, *p* = 1; FRO: V = 12, *p* = 0.777), and it raised above chance level in the last two trials (FXO/1: V = 153, *p* < 0.001; FXO/2: V = 152, *p* = 0.001; FLO: V = 171, *p* < 0.001; FRO: V = 49.5, *p* = 0.013). Therefore, in adult participants, performance improves during the test phase for all grammars, though less sharply for the free order grammar.

### Children

The results of our experiments with kindergarten children are different from the pattern found in adults. In Experiments 3 and 4, children were not able to recognize strings from the fixed order grammars FXO/1 and FXO/2, although they had been exposed to each of these grammars, respectively: performance was at chance in both cases (**Figures 2C,D** and **Table 3**). However, children were able to recognize strings from the flexible order grammar FLO, and even from the free order grammar FRO, as evidenced by above-chance performance in both cases (**Figures 2C,D** and **Table 3**). A between-groups ANOVA revealed a main effect of Grammar [η^2^= 0.12, *F*(3,92) = 4.498, *p* = 0.005], independent of the first language of children [Grammar × L1: η^2^= 0.04, *F*(2,92) = 2.278, *p* = 0.108]. In line with our predictions, children were able to recognize strings compatible with the typologically attested patterns (flexible and free order grammars), while they failed with strings following a typologically deviant pattern.

As in adults, we analyzed children’s changing performance during the test phase. We observed an increase in the number of correct responses over trials in the flexible order grammar group [**Figure 3F**; FLO, *R*^2^= 0.06, η^2^= 0.066, *F*(1,174) = 12.21, *p* = 0.0006] and in the free order grammar group [**Figure 3H**; FRO, *R*^2^= 0.033, η^2^= 0.038, *F*(1,222) = 8.671, *p* = 0.0036], but only marginally in the fixed order group FXO/1 [**Figure 3E**; *R*^2^= 0.031, η^2^= 0.037, *F*(1,174) = 6.606, *p* = 0.01], and not in FXO/2 [**Figure 3G**; *R*^2^= -0.004, η^2^= 0.0003, *F*(1,222) = 0.068, *p* = 0.794]. In both flexible and free order grammar groups, performance was at chance in the first two trials (FLO, *V* = 39, *p* = 1; FRO, *V* = 16, *p* = 0.78), and it gradually improved, raising above chance in the last two trials (FLO, *V* = 104, *p* = 0.005; FRO, *V* = 178.5, *p* = 0.002). In Experiment 3, in the fixed order group, performance is initially below chance (FXO/1, *V* = 22.5, *p* = 0.035), and reaches chance at the end of the test phase (*V* = 42, *p* = 0.39). In Experiment 4, instead, performance in the fixed order group is at chance both at the beginning of the test phase (FXO/2, *V* = 32.5, *p* = 0.59) and at the end of it (*V* = 37.5, *p* = 0.3). These results suggest that learning during the test phase was easier with grammars conforming to the attested typological pattern (FLO and FRO). The fact that children perform at chance at the beginning of the test phase does not license the inference that they do not learn during exposure, or that exposure has no effect on learning. It does suggest, however, that interaction and feedback are beneficial, and possibly necessary, either for learning to occur, or for bringing out the effects of learning accumulated during exposure.

## Discussion

We investigated whether adults and kindergarten children recognize strings from artificial finite-state grammars with fixed, flexible and free word order, and two word classes: shorter, more frequent words, or F-words, and longer, less frequent words, or C-words. The critical test was whether participants would recognize strings with the same grammatical structure as strings from the exposure set, now using a different vocabulary. Our predictions were all borne out. Adults could recognize strings from the target grammar, if a set of underlying regularities could be identified: i.e., they succeeded with fixed and flexible order strings, and failed with free order strings. In contrast, children could only recognize strings when they conformed to the typological pattern: they succeeded with free and flexible order, and failed with fixed order. Here, we discuss these results on the basis of two assumptions. First, learning did occur, as attested by changes in recognition performance. However, learning here may be limited to the *extraction of structural properties of strings*. We do not assume that participants were able to *extend those properties to strings of arbitrary length*, i.e., that they effectively acquired the target grammars. Note that, in general, based on observations involving a finite number of strings, it is logically impossible to prove that learners acquire the target grammar, as opposed to an extensionally equivalent grammar for the relevant string sets. Second, we assume that the extraction of structural properties of strings is a necessary step toward grammar induction: grammar rules are discoverable if and only if the structures to which those rules apply are correctly identified and represented as such. Our results therefore show that structure extraction is constrained in kindergarten children. But because structure extraction is a necessary component of grammar learning, our data also show that grammar learning itself is constrained.

Before we attempt an interpretation of these results, we must address alternative explanations, identifying the sources of learning ease or difficulty in the surface features of strings, rather than in the underlying structure. One possibility is that fixed order strings are ‘simpler’ than flexible and free order strings, thus fixed order strings would not engage the children’s attention or memory to a degree sufficient for learning to initiate or occur. However, we can exclude that strings from any grammar were either more or less complex than strings from any other. Based on formal measures of complexity applied to the relevant strings (**Table 1**), one can show that strings of the same length are comparable in complexity across grammars (see Materials and Methods). This desirable property scales up to strings of increasing length, including strings that were not used in our experiments. Therefore, string complexity is not a factor that determines whether attention or memory are allocated to different extents in attempting to learn different grammars. But perhaps other factors are at play than string complexity, that determine whether strings are *perceived* as being more or less engaging for learners. One idea is that the type or the frequency of local transitions, either between words of the same category (FF or CC), or between words of different categories (FC or CF), provide cues to the learner, such that a grammar with *fewer* such cues may be *harder* to learn. However, there is no systematic pattern of transition frequencies across grammars that explains the results reported above (**Table 1**). In Experiment 3, FLO has more across-category transitions (FC and FC) than FXO/1, which may explain why FXO/1 is harder for children, however, in Experiment 4 FXO/2 has more such transitions, yet it is harder for children. Similarly, in Experiment 4, FRO has more within-category transitions (FF and CC) than FXO/2, perhaps rendering it is easier to learn, but in Experiment 3 FXO/1 has more such transitions, yet it is harder. In brief, neither string complexity nor word transition patterns in our stimuli predict which grammars are harder to learn. Another idea is that fixed order grammars may be harder to learn, because they are implemented in more complex machines structurally: indeed, the automata generating and recognizing fixed order strings have 4 states vs. 2 in the automata for flexible and free order grammars. This explanation, too, appears unconvincing, not so much because a difference between 4 vs. 2 states is too small to have an effect on learning (which it probably is), but because, if it did, one should expect the same effect on adult learners, which was not the case. Yet another alternative explanation would suggest that children did not initially understand the task. The feedback children received upon seeing the location of the puppet inside each box may have simply clarified the task, rather than provided them with information about the grammar. But even if that was the case, there would be differences in learning the different grammars (structure extraction), which this kind of alternative explanation does not address. Finally, there may be an alternative explanation of learning performance in Experiment 3. The FXO/1 grammar involves a *non-adjacent dependency* between 2 F-words, while the FLO grammar involves an *adjacent dependency*. Non-adjacent dependencies may be harder to learn in general, and adults might be better at initial stages of L2 learning than children. If we see an artificial language as a special case of L2 learning we may conclude that adults are better or faster than children at tracking complex statistical regularities, as those involved in non-adjacent dependencies, within a limited amount of time. However, this cannot explain data from the FXO/2 grammar in Experiment 4, where the recurring pattern (FCF…) is in fact an *adjacent* dependency, spanning 3 words.

We designed our artificial grammars so that two were possible images of typological patterns of distribution of function and content words, and two were violations of those. Moreover, we observed that learning in children is easier for the grammars that follow the typological pattern. Nonetheless, we cannot infer from these premises that these grammars (or strings) are easier for children *because* they conform to the typological pattern. Our conclusion is rather more modest, namely that, in the present study, we were unable to falsify the hypothesis that learning constraints in children mirror typological patterns. However, we do provide more direct evidence for (a) the existence of developmental constraints on learning artificial grammars, and specifically on the *extraction of structure from strings*, whatever the exact nature of these constraints, and for (b) the role of interaction and feedback in learning. We briefly discuss these two points.

Possible differences in learning processes between adults, infants and children have attracted considerable attention (Hudson Kam and Newport, 2009; Fava et al., 2011; Finn et al., 2014). What might account for such differences, specifically for the different responses between adults and children observed in our study? One hypothesis is that learning constraints specific to language undergo maturational decay, or that language learning abilities decline owing to the emergence or expansion of non-linguistic cognitive abilities (Newport, 1990; Ramscar and Gitcho, 2007) or to the effects of neurobiological constraints on learning syntactic categories (Lenneberg, 1967). It has also been proposed that children and adults rely on different processes and implicit strategies for abstract rule extraction, or for building linguistic competence, more generally (Newport, 1990; Hudson Kam and Newport, 2005, 2009). In traditional terms, one may view our results as being consistent with a putative ‘critical period,’ after which the ability to learn any additional languages changes or declines (Lenneberg, 1967; Newport, 1990). Its existence has been contested, however. Already Piaget (1926) had argued that cognitive differences between adults and children, including language learning, were rather a result of the overall maturation of the brain, plus the fact that, for children, learning a language is part of an attempt at understanding the surrounding world. Therefore, adults simply rely on *different* learning mechanisms than children, and those are not necessarily more or less constrained than in children (Scovel, 2000). In sum, although our results point to the existence of developmental constraints *on* language learning, we suspend judgment on whether such constraints are, in some relevant sense, *for* language learning, and on whether our experimental results may be taken to support critical period hypotheses.

Our results shed some light on the relations between implicit learning and social interaction during language acquisition. One may argue that there were actually *two* training sessions in our experiment: one, which consisted of passive listening (‘exposure phase’), and another, consisting of supervised learning (‘test phase’). We provide evidence that children (also) learn during the test phase, and that supervised, interactive learning appears important to drive recognition performance above chance levels. We cannot exclude that children learn *only* during the test phase. However, (a) this possibility would not undermine the idea that learning is constrained in ways compatible with typological patterns, and (b) it would be a deceptively parsimonious account, which presupposes a mechanism whereby children can learn to discriminate strings of different length and structure *simultaneously and in few trials* (six strings were presented in each trial), given the design of our test phase. This kind of fast, parallel learning in children is to our knowledge not documented in the literature. On the other hand, the idea that implicit learning during the exposure phase is *sufficient* to produce learning effects is positively ruled out by our data. The middle ground position seems most plausible here: although mere exposure to speech stimuli might suffice for infants to extract statistical regularities in early language acquisition (Saffran et al., 1996, 1999; Gómez and Gerken, 2000), implicit learning and interaction (with feedback) seem just as important to produce observable learning effects, in particular when strings instantiate complex structural principles. Recent research has suggested that ‘innate’ learning biases are amplified in the course of language transmission across generations (Kirby et al., 2008; Thompson et al., 2016), in agreement with the hypothesis that biases and constraints play out when speakers and learners from different generations interact. Some of the latest work in this field is introducing peer interaction directly into laboratory or computational models of language transmission (Kirby et al., 2015; Moreno and Baggio, 2015; Nowak and Baggio, 2016; Lumaca and Baggio, 2016, 2017). Language learning can therefore be viewed as a complex process, where social learning and cultural transmission are crucial for producing, amplifying and stabilizing the effects of developmental constraints on language structure.

## Author Contributions

IN and GB developed the study concept, designed the experiments, analyzed the data and wrote the manuscript. IN constructed the stimuli and collected the data. All authors approved the final version of the manuscript.

## Conflict of Interest Statement

The authors declare that the research was conducted in the absence of any commercial or financial relationships that could be construed as a potential conflict of interest.
