# Supplementary material for: Developmental Constraints on Learning Artificial Grammars with Fixed, Flexible and Free Word Order
Source: Front Psychol. 2017 Oct 17;8:1816. doi: 10.3389/fpsyg.2017.01816 (PMC5651074; doi:10.3389/fpsyg.2017.01816)
Supplement: Supplementary file 1 [file Supplementary_Material.PDF]

Supplementary material: Sample stimuli

FXO/1

UL GANOF PE (FCF)
EN NOFO PLUFO RI (FCCF)
OF CUBE PEGO TUMU BA (FCCCF)
OM RAGHI DIMUT FIRI MUSU GO (FCCCCF)

RI BOFI AZ (FCF)
OM RANO TAGA OF (FCCF)
CU UNOP SAMA MEFU PE (FCCCF)
EN PALO SASNE MOGO ALFON BA (FCCCCF)

EN GRIBU OF (FCF)
BA PILI BAMAR CU (FCCF)
GO LIPU ELAT CUPIL PE (FCCCF)
UL SAGUF EDER POME BITA RI (FCCCCF)

BA TUMU GO (FCF)
UL BIRI GHEDO EN (FCCF)
OM BURUM POME GUSNU PE (FCCCF)
OF UMAR GOPI RELAL BEDU RI (FCCCCF)

RI SAZO OF (FCF)
EN OTOD DIPI UL (FCCF)
GO TEMU RAMU FAVO CU (FCCCF)
BA LOGHI SADI ROFA LAFO OM (FCCCCF)

GO NECON BA (FCF)
OF RIGO PITAM UL (FCCF)
PE LOBAT MEFU DANA OM (FCCCF)
EN RIMRA CREGA SADI TEDAN CU (FCCCCF)

CU ZALO AZ (FCF)
RI FECHE BAMAR EN (FCCF)
OF MUSUT ELUD SOPA GO (FCCCF)
UL DEMAG NEFA AREM PALOT PE (FCCCCF)

Supplementary material: Sample stimuli

FXO/2

UL GANOF PE (FCF)
EN NOFO RI PLUFO (FCFC)
OF CUBE BA PEGO TUMU (FCFCC)
OM RAGHI GO DIMUT FIRI MUSU (FCFCCC)

RI BOFI AZ (FCF)
OM RANO OF TAGA (FCFC)
CU UNOP PE SAMA MEFU (FCFCC)
EN PALO BA SASNE MOGO ALFON (FCFCCC)

EN GRIBU OF (FCF)
BA PILI CU BAMAR (FCFC)
GO LIPU PE ELAT CUPIL (FCFCC)
UL SAGUF RI EDER POME BITA (FCFCCC)

BA TUMU GO (FCF)
UL BIRI EN GHEDO (FCFC)
OM BURUM PE POME GUSNU (FCFCC)
OF UMAR RI GOPI RELAL BEDU (FCFCCC)

RI SAZO OF (FCF)
EN OTOD UL DIPI (FCFC)
GO TEMU CU RAMU FAVO (FCFCC)
BA LOGHI OM SADI ROFA LAFO (FCFCCC)

GO NECON BA (FCF)
OF RIGO UL PITAM (FCFC)
PE LOBAT OM MEFU DANA (FCFCC)
EN RIMRA CU CREGA SADI TEDAN (FCFCCC)

CU ZALO AZ (FCF)
RI FECHE EN BAMAR (FCFC)
OF MUSUT GO ELUD SOPA (FCFCC)
UL DEMAG PE NEFA AREM PALOT (FCFCCC)

Supplementary material: Sample stimuli

FLO

GANOF UL OLBO (CFC)
NOFO EN PLUFO RI (CFCF)
CUBE OF PEGO BA TUMU (CFCFC)
RAGHI OM DIMUT GO FIRI MUSU (CFCFCC)

BOFI RI DEPO (CFC)
RANO OM TAGA OF (CFCF)
UNOP CU SAMA PE MEFU (CFCFC)
PALO EN SASNE BA MOGO ALFON (CFCFCC)

GRIBU EN SOPA (CFC)
PILI BA BAMAR CU (CFCF)
LIPU GO ELAT PE CUPIL (CFCFC)
SAGUF UL EDER RI POME BITA (CFCFCC)

TUMU BA ZAMO (CFC)
BIRI UL GHEDO EN (CFCF)
BURUM OM POME PE GUSNU (CFCFC)
UMAR OF GOPI RI RELAL BEDU (CFCFCC)

SAZO RI EMAG (CFC)
OTOD EN DIPI UL (CFCF)
TEMU GO RAMU CU FAVO (CFCFC)
LOGHI BA SADI OM ROFA LAFO (CFCFCC)

NECON GO EMER (CFC)
RIGO OF PITAM UL (CFCF)
LOBAT PE MEFU OM DANA (CFCFC)
RIMRA EN CREGA CU SADI TEDAN (CFCFCC)

ZALO CU FORE (CFC)
FECHE RI BAMAR EN (CFCF)
MUSUT OF ELUD GO SOPA (CFCFC)
DEMAG UL NEFA PE AREM PALOT (CFCFCC)

Supplementary material: Sample stimuli

FRO

UL GANOF OLBO (FCC)
NOFO EN RI PLUFO (CFFC)
CUBE PEGO OF TUMU BA (CCFCF)
OM RAGHI DIMUT GO FIRI MUSU (FCCFCC)

RI BOFI DEPO (FCC)
RANO OM OF TAGA (CFFC)
UNOP SAMA CU MEFU PE (CCFCF)
EN PALO SASNE BA MOGO ALFON (FCCFCC)

EN GRIBU SOPA (FCC)
PILI BA CU BAMAR (CFFC)
LIPU ELAT GO CUPIL PE (CCFCF)
UL SAGUF EDER RI POME BITA (FCCFCC)

BA TUMU ZAMO (FCC)
BIRI UL EN GHEDO (CFFC)
BURUM POME PE GUSNU OM (CCFCF)
OF UMAR GOPI RI RELAL BEDU (FCCFCC)

RI SAZO EMAG (FCC)
OTOD EN UL DIPI (CFFC)
TEMU RAMU GO FAVO CU (CCFCF)
BA LOGHI SADI OM ROFA LAFO (FCCFCC)

GO NECON EMER (FCC)
RIGO OF UL PITAM (CFFC)
LOBAT MEFU PE DANA OM (CCFCF)
EN RIMRA CREGA CU SADI TEDAN (FCCFCC)

CU ZALO FORE (FCC)
FECHE RI EN BAMAR (CFFC)
MUSUT ELUD OF SOPA GO (CCFCF)
UL DEMAG NEFA PE AREM PALOT (FCCFCC)
